# Inflammatory Pain and Corticosterone Response in Infant Rats: Effect of 5-HT1A Agonist Buspirone Prior to Gestational Stress

**DOI:** 10.1155/2013/915189

**Published:** 2013-03-31

**Authors:** Irina P. Butkevich, Viktor A. Mikhailenko, Tat'yana R. Bagaeva, Elena A. Vershinina, Anna Maria Aloisi, Vladimir A. Otellin

**Affiliations:** ^1^Laboratory of Ontogeny of the Nervous System, I. P. Pavlov Institute of Physiology, Russian Academy of Sciences, Nab. Makarova 6, St. Petersburg 199034, Russia; ^2^Laboratory of Experimental Endocrinology, I. P. Pavlov Institute of Physiology, Russian Academy of Sciences, Nab. Makarova 6, St. Petersburg 199034, Russia; ^3^Department of Applied Mathematics, I. P. Pavlov Institute of Physiology, Russian Academy of Sciences, Nab. Makarova 6, St. Petersburg 199034, Russia; ^4^Department of Physiology, University of Siena, Neuroscience and Applied Physiology Section, Via Aldo Moro 2, 53100 Siena, Italy

## Abstract

Our researches have shown that gestational stress causes exacerbation of inflammatory pain in the offspring; the maternal 5-HT1A agonist buspirone before the stress prevents the adverse effect. The serotonergic system and hypothalamo-pituitary-adrenal (HPA) axis are closely interrelated. However, interrelations between inflammatory pain and the HPA axis during the hyporeactive period of the latter have not been studied. The present research demonstrates that formalin-induced pain causes a gradual and prolonged increase in plasma corticosterone level in 7-day-old male rats; twenty-four hours after injection of formalin, the basal corticosterone level still exceeds the initial basal corticosterone value. Chronic treatments of rat dams with buspirone before restraint stress during gestation normalize in the offspring pain-like behavior and induce during the acute phase in the formalin test the stronger corticosterone increase as compared to the stress hormonal elevation in animals with other prenatal treatments. Negative correlation between plasma corticosterone level and the number of flexes+shakes is revealed in buspirone+stress rats. The new data enhance the idea about relativity of the HPA axis hyporeactive period and suggest that maternal buspirone prior to stress during gestation may enhance an adaptive mechanism of the inflammatory nociceptive system in the infant male offspring through activation of the HPA axis peripheral link.

## 1. Introduction

Interrelations between the serotonergic system and hypothalamo-pituitary-adrenal (HPA) axis determine the formation of mechanisms of stress adaptation [1–6]. Pain is a stress and therefore can activate the HPA axis [7–15]. In this case, inflammatory pain is still not clearly understood, and the data available are inconsistent [9, 14–17]. In a widely used model of inflammatory pain, the formalin test, activation of the HPA axis with the nociceptive stimulus formalin has been shown on adult awake rodents and differences in the dynamics of release of ACTH and corticosterone in response to pain impact found [8, 9, 17–19]. The HPA axis during the postnatal development goes through the period of hyporesponsiveness, which extends from the second to fourteenth postnatal days and is characterized by a low level of the response of adrenals to many stress stimuli [20]. Investigations of formalin-induced pain effects on the HPA axis in infant rats could elucidate unexplored previously interrelations between the tonic nociceptive and stress systems during the period of hyporesponsiveness of the latter. We revealed for the first time that prenatal stress induces strengthening inflammatory pain-related response in the formalin test and decrease of adaptive capacities in infant rats; chronic injections of an agonist of serotonin (5-HT) receptors 1A (5-HT1A) buspirone to dams prior to stress during gestation cancel the adverse consequences of the stress in the offspring [21]. In prenatally stressed individuals, abnormalities in the HPA axis function [22] and neurotransmitter systems including the serotonergic one [23] were shown. 

Serotonin acts as a growth factor in early cell division, migration, and differentiation in the brain specifically in development of the serotonergic system [24–27]. Many of regulator influences on developing neurons 5-HT mediates through presynaptic 5-HT1A autoreceptors in the raphe nuclei [28, 29]. Later 5-HT and 5-HT1A receptors take part in many kinds of behavior. The serotonergic system plays an important role in pain transmission, its processing and regulation [30–33]. Buspirone, serotoninergic anxiolytic and antidepressant, mediates its effect through the serotonergic system and the HPA axis. There are synergistic interrelations between these systems impaired in prenatally stressed individuals [23, 34–36]. A peculiar mechanism of buspirone action has not been completely understood; it is also true for its analgesic effect. Studies of effects of buspirone, an agonist of presynaptic and a partial agonist of 5-HT1A receptors, on the nociceptive system are limited, and the results obtained do not coincide [37, 38]. Prenatal effect of buspirone on the nociceptive system has not been studied until our researches. Activation of the antinociceptive descending serotonergic system and the decrease in hyperactivity of the HPA axis are considered as potential mechanisms of analgesic action of antidepressants. Activating effects of buspirone on the HPA system are found in adult persons [39]. It may be suggested that the period of hyporesponsiveness of the HPA, which is characterized by a low level of reaction of adrenals in response to many stress stimuli [20], will allow to prevent activating influences of buspirone on the HPA axis. 

The aim of our work was to study effects of maternal buspirone prior to stress during gestation on the dynamics of the inflammatory pain-like behavior and stress response of corticosterone during the formalin test in the infant male rat offspring and also to evaluate correlation between pain-like and hormonal parameters.

## 2. Materials and Methods

### 2.1. Animals

All experimental procedures were approved by the Local Ethics Committee for Animal Experiments of the I. P. Pavlov Institute of Physiology and followed the guidelines published by the Committee for Research and Ethical Issues of the IASP on ethical standards for investigations of experimental pain in animals.

Adult female rats and male rats (Wistar) at the age of 90 days were obtained from the vivarium of the I. P. Pavlov Institute of Physiology RAN, St. Petersburg, Russia. Two days after adaptation, the rats were mated. The days of insemination and delivery were considered as gestational day (GD) 0 and postnatal day (PD) 0, respectively. All animals were maintained at constant temperature (20–22°C) under the standard light-dark cycle (8.00 AM–8.00 PM) with unrestricted access to food and water. Seventeen rat dams (controls) were not exposed to any impacts during gestation. The equal number of remaining dams (*n* = 68) was randomly treated with the 5-HT1A agonist buspirone (buspirone hydrochloride, Sigma, 3 mg/kg, 1 mL, i.p. at 9 AM) or with injection of saline (control animals from the same litters, in the same conditions of injections) from GD9 to GD21. A half of the treated rats from each group were randomly exposed to restraint stress for 60 min (in 5 min after buspirone injection) from GD15 to GD21. All influences on gestational females were identical to those used in our previous study [21]. The dose of buspirone was sufficient for inducing an anxiolytic effect in adult rats [40] and did not exceed the dose used for pregnant rat dams to protect the fetal serotonergic system against damaging effects of in utero ethanol exposure [41]. It should be noted that such dose of buspirone was not able to implicate dopamine and norepinephrine in the mediation of buspirone effects [42, 43]. Litters were called to 8 pups (4 females and 4 males, as far as possible) in 48 hours after birth. In the study, 7-day-old males born to the dams with the above-mentioned treatments during gestation and to control dams were used; females and remaining males were used in other researches. There were 245 males offspring of control, saline, saline + stress, buspirone, and buspirone + stress dams in the study with formalin injection (about 6–8 males per a group, no more than 3 animals from one dam); in addition, 80 male rats from the same litters were used as control for the formalin, with saline injection into the hind limb.

### 2.2. Experimental Formalin-Induced Inflammatory Pain in Infant Male Rats

Formalin test is widely used for evaluation of tonic inflammatory pain and analgesic effects of various pharmacological drugs [9, 44–47]. Flexing and shaking behaviors are the specific expression of inflammatory pain-related behavior in the formalin test in both infant and adult rats [44, 45, 48, 49]; we used the formalin test as previously described [21]. The formalin test allows evaluating acute nociception (the first phase, 5–10 min after formalin injection), tonic persistent nociception (the second phase about 30–40 min), and functional activity of the descending serotonergic inhibitory system (the interphase about 3–10 min). The second phase appears during postnatal development when the descending serotonergic inhibitory system matures [45, 46]. Characteristics of the phases depend on many factors including age and sex [50]. 

Each male rat was taken from the nest, injected intraplantarly to the left hindpaw with formalin solution (2.5%, 10 *μ*L), and placed singly in a warm (25°C) chamber (25 × 20 × 10 cm) with transparent glass walls encircled by mirrors to improve the observation of the animal's behavior [21, 51]. The number of flexes + shakes was recorded using a computer program that allows recording, quantifying, and analyzing the pain-related behavior. In each group of the males, the number of flexes + shakes was averaged for 3, 9, 21, 30, and 60 min after formalin injection. Each animal was used only once.

### 2.3. Corticosterone Determination in Infant Male Rats

Blood samples were collected by decapitation in the rats with different prenatal treatments and in controls at 09:00-10:00 before and 24 hours after the formalin test for determination of basal plasma corticosterone levels. During the formalin-induced pain, blood sampling by decapitation occurred at 3, 9, 21, 30, and 60 min after formalin injection. The blood samples were centrifuged and the plasma was kept at −20°C no more than a week. The plasma corticosterone (SIGMA-ALDRICH, USA) levels (*μ*g/dL) were measured by microfluorometry [52]. 

### 2.4. Statistical Analysis

Data are presented as mean ± S.D. Formalin-evoked flexing + shaking, and corticosterone responses were analyzed by two-way ANOVA, with treatment (control, saline, saline + stress, buspirone, and buspirone + stress) and time as factors. Behavioral and corticosterone responses during 3, 9, 21, 30, and 60 minutes were separately evaluated. Comparisons between the basal levels and the data over time as well as comparisons between groups with different types of treatment were conducted using tests of simple effects. Besides pairwise comparisons, *t*-test and Mann-Whitney test were performed. Pearson and Spearman Correlations were calculated to estimate relationships between behavioral and hormonal variables. For all tests, *P* < 0.05 was considered to be statistically significant.

## 3. Results

**Figure 1:**
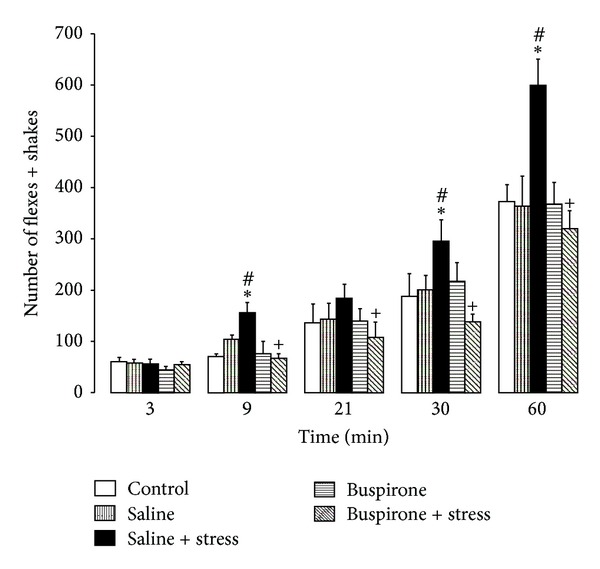
Pain-like responses recorded over different periods of time (3, 9, 21, 30, and 60 min) after injection of formalin (mean ± SEM) in 7-day-old male rats with different prenatal treatments. *P < 0.05 different from saline; ^+^ *P* < 0.05 different from saline + stress; ^#^ *P* < 0.05 different from controls.

Two-way ANOVA applied to pain-like responses (Figure 1) resulted in a significant effect of the factor prenatal treatments (F(4,114) = 5.094, *P* = 0.001); (F(3,114) = 56.545, *P* < 0.001). Tests of simple effects showed a significant increase in the number of flexes + shakes at 9, 30, and 60 min after formalin injection in saline + stress as compared to saline (*P* < 0.05, *P* < 0.05, and *P* = 0.014, resp.) and in saline + stress as compared to the control (*P* = 0.002, *P* = 0.03, resp.) (Figure 1). Tests of simple effects showed a decrease in the number of flexes + shakes at 9, 21, 30, and 60 min after formalin injection in buspirone + stress as compared to saline + stress (*P* = 0.002, *P* < 0.05, *P* < 0.05, and *P* = 0.002, resp.) (Figure 1).

**Figure 2:**
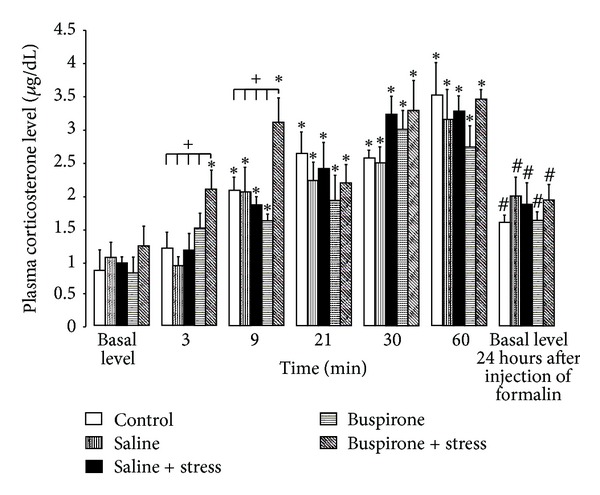
Basal levels of corticosterone and plasma corticosterone levels (*μ*g/dl) determined 3, 9, 21, 30, and 60 min after injection of formalin (mean ± SEM) in 7-day-old male rats with different prenatal treatments. *P < 0.05 different from the basal level; ^+^ *P* < 0.05 different from the control, saline, saline + stress, and buspirone; ^#^ *P* < 0.05 different from the basal level before injection of formalin.

Two-way ANOVA applied to the level of plasma corticosterone (Figure 2) resulted in a significant effect of the factor prenatal treatments (F(4,170) = 2.706, *P* = 0.002) and time (F(5,170) = 22.574, *P* < 0.001). The significant effects of factor prenatal treatment on dependent variable corticosterone were revealed at 3 and 9 min (F(4,170) = 2.322, *P* < 0.05; F(4,170) = 3.634, *P* < 0.007, resp.). Tests of simple effects showed the corticosterone level at 3 min after formalin injection was higher than basal level (*P* = 0.037) in buspirone + stress males; during the following time periods (9, 21, 30, and 60 min), corticosterone was higher than basal level in animals with all prenatal treatments (*P* < 0.05) (Figure 2). Tests of simple effects (pairwise comparisons) or/and Mann-Whitney test showed that only in buspirone + stress males at 3 and 9 min after formalin injection, the corticosterone level was higher than similar hormonal level in animals with all different prenatal treatments (*P* < 0.05). During the following time course of formalin-induced pain, there were no differences in the stress level of hormone between animals with different prenatal treatments. Tests of simple effects (pairwise comparisons) or/and Mann-Whitney test showed that in the course of inflammatory pain, the level of plasma corticosterone gradually increased (F5,170) = 22.574, *P* < 0.001) and to the end of the formalin test (at 60 min) was significantly higher than basal level (*P* = 0.001). Pairwise comparisons showed that basal level 24 h after the formalin test was greater than that prior to the formalin test (*P* = 0.001) in animals with all prenatal treatments (*P* < 0.05) (Figure 2).

There were no significant differences in indices under study in buspirone, saline, and control animals.

Correlation between plasma corticosterone level and the number of flexes + shakes was revealed in buspirone + stress male rats at 3 (−*r* = 0.925, *P* = 0.008), 9 min (−*r* = 0.937, *P* = 0.002), and 60 min (−*r* = 0.690, *P* = 0.05) and in control rats at 3 min (−*r* = 0.90, *P* = 0.037) after injection of formalin. 

Injection of saline to the left hindpaw (controls for formalin injection) resulted in a few weak flexes + shakes during some first minutes after injection only in prenatally stressed males. Corticosterone response to pain was a specific reaction; in control animals, an increase in the plasma corticosterone in response to the procedure of saline injection into the paw was less prolonged (no more than 30 min) and did not exceed the value of corticosterone response to formalin-induced pain (the data are not shown in the table). 

## 4. Discussion

The dynamics of corticosterone stress response to inflammatory pain and participation of 5-HT1A receptors in it were investigated in the present study in 7-day-old male rats with various prenatal treatments. Evidence of increased formalin-induced pain in prenatally stressed animals is in agreement with the data that we obtained earlier [21]. Chronic treatments of rat dams with the 5-HT1A agonist buspirone prior to stress during gestational period increased resistance of the tonic nociceptive system normalizing behavior in the inflammatory pain model and changed the time course of stress corticosterone response to formalin-induced pain in the offspring.

Before our studies, in a widely accepted model of inflammatory pain, the formalin test, it was demonstrated in adult awake rats that the nociceptive stimulus formalin induced activation of the HPA axis and increased concentration of ACTH and corticosterone [9, 13, 19]. Interestingly, the authors that found the peak of the corticosterone release at 30 min and its restored level at 80 min after formalin injection concluded that the resulting release of corticosterone is not antinociceptive as neither adrenalectomy nor high-dose dexamethasone changed behavioral nociceptive responses [9]. It is worthy to note that peaked time in release of corticosterone in response to the formalin test as well as the time of the hormonal restoration level after formalin injection vary according to the authors from the 15–60 min to 60–120 min, respectively [9, 13, 19]. These differences may be attributed to peculiarities of the formalin test. The behavioral response in the formalin test, represented by acute and tonic phases of different chemical nature, depends on concentrations and volumes of formalin solution, a place of its injection, temperature in the room, a strain of rats, and conditions of experimental performances [9, 50, 53, 54]. These factors determine involvement in the response of various mediators influencing the intensity and dynamics of release of corticosterone. 

Our study is the first to evaluate the dynamics of corticosterone release in conditions of inflammatory pain in infant rats during the hyporesponsive period of the HPA axis [20]. The new data obtained testify that inflammatory formalin-induced pain evokes the stress response of corticosterone in male rats during the hyporesponsive period of the HPA axis. This reaction is a specific reaction to pain; in control animals, an increase in the plasma corticosterone in response to the procedure of saline injection into the paw was less prolonged and weaker than the hormonal response to formalin-induced pain. We have revealed that the characteristic feature of the dynamics of corticosterone response to inflammatory pain in infant rats is a gradual increase of hormonal release in the formalin test, so to the end of the response the level of corticosterone considerably exceeded its initial level. Most importantly, the results indicate that 24 hours after the formalin test, the corticosterone level still exceeds the basal corticosterone value before the formalin test in males of all the groups under study. This fact cannot be associated with an increase in corticosterone basal level in intact 8-day-old male rats as compared to that in 7-day-olds, as available data and our own results indicate equal value in the basal level of plasma corticosterone in 5–8-day-old male rats that were not exposed to any prenatal impacts [55]. Based on these results, we conclude that the peripheral link of the HPA axis responds to inflammatory pain in the formalin test in infant rats with a prolonged reaction. Experimental data reported here enhance the idea of relativity of hyporesponsive period of the HPA axis [20]. Up to now, there has not been any detailed work done to find a clear explanation for this period in the development of the HPA axis. 

The results obtained provide new important information that maternal 5-HT1A agonist buspirone prior to stress during gestation induces in the offspring during the acute phase in the formalin test the stronger corticosterone increase as compared to the stress hormonal elevation in animals with other prenatal treatments. In the following time periods of formalin-induced pain, the animals with different prenatal treatments do not show significant differences in stress corticosterone level. Negative correlation revealed between the corticosterone concentration and the number of flexes + shakes during the first nine minutes after injection of formalin in buspirone + stress male rats is noteworthy. These results suggest that activation of the corticosterone release via 5-HT1A receptors may facilitate some adaptive mechanisms associated with a decrease of inflammatory pain in buspirone + stress rats.

There are multiple pathways through which 5-HT and its agonists may stimulate the HPA axis [56]. It is shown that formalin activates ascending ways to the HPA [57]. The chemical stimulus formalin induces appearance of “inflammatory soup” from various chemical substances including 5-HT released from platelets and also activation of neutrophils and leucocytes that produce proinflammatory cytokines IL-6 [17]. Cytokines contribute to the increase in ACTH and corticosterone [17] and to the exacerbation of nociceptive processing [58]. Interaction between the HPA axis and 5-HT system would be dependent on concentration of 5-HT released from platelets during inflammation which reaches the central nervous sites, but this question is poorly known. Both systems are highly plastic during maturation [6], and prenatal stress impairs interaction between the HPA axis and 5-HT system and alters their functional activity in adults [2, 35]. The expression of 5-HT1A receptors is found during the initial stages of prenatal development of the hippocampus [59] and prenatal stress impairs their development [60]. There is evidence that buspirone penetrates through the placental and blood brain barriers [61] and is able to exert the protective effects presumably through its ability to overcome the deficit of fetal serotonin and to stimulate fetal 5-HT1A receptors [62]. Further studies are needed to evaluate influences of maternal buspirone prior to stress during gestation to the HPA axis response during the inflammatory pain immediately after finishing the period of responsiveness in the HPA axis development. Thus, new data indicate an important role of 5-HT1A receptors in the development of close relationships between the HPA axis and tonic nociceptive system that mediate adaptation of organism to extreme conditions.

## 5. Summary

The formalin-induced pain causes a gradual and prolonged increase in plasma corticosterone level during the persistent pain-like behavior in 7-day-old male rats. Chronic treatments of rat dams with buspirone before restraint stress during gestation normalize in the offspring inflammatory pain behavior and induce during the acute phase in the formalin test the stronger corticosterone increase as compared to the stress hormonal elevation in animals with other prenatal treatments. Buspirone + stress rats display the negative correlation between plasma corticosterone level and the number of flexes + shakes. Thus, the new data enhance the idea about relativity of the HPA axis hyporeactive period and suggest that maternal buspirone prior to stress during gestation may enhance an adaptive mechanism of the inflammatory nociceptive system in the infant male offspring through activation of the HPA axis peripheral link.
